# Supplementary material for: Evaluation of the Potential Risk Posed by Emerging Yr5-Virulent and Predominant Races of Puccinia striiformis f. sp. tritici on Bread Wheat (Triticum aestivum L.) Varieties Grown in Türkiye
Source: J Fungi (Basel). 2025 Aug 29;11(9):635. doi: 10.3390/jof11090635 (PMC12470476; doi:10.3390/jof11090635)
Supplement: Supplementary file 1 [file jof-11-00635-s001.zip › jof-3775984-supplementary.pdf]

Evaluation of the Potential Risk Posed by Emerging *Yr5*-Virulent and Predominant Races of *Puccinia striiformis* f. sp. *tritici* on Bread Wheat (*Triticum aestivum* L.) Varieties Grown in Türkiye

Kadir Akan [1], Ahmet Cat [2], Medine Yurduseven [3,4], Yesim Sila Tekin [3,5], Mehmet Zahit Yeken [6] and Mehmet Tekin [7,*]

[1]Department of Plant Protection, Faculty of Agriculture, Kırşehir Ahi Evran University, Kırşehir, Türkiye; kadir_akan@hotmail.com

[2]Department of Plant Protection, Faculty of Agriculture, Siirt University, Siirt, Türkiye; ahmetcat@siirt.edu.tr

[3]Department of Field Crops, Institute of Natural and Applied Sciences, Akdeniz University, Antalya, Türkiye

[4]Argeto Vegetable Seeds Co. Antalya, Türkiye; yurdusevenmdn07@gmail.com

[5]Merkez Anadolu Kimya Co., Antalya, Türkiye; ysilatekin@gmail.com

[6]Department of Field Crops, Faculty of Agriculture, Bolu Abant İzzet Baysal University, Bolu, Türkiye; yekenmehmetzahit@gmail.com

[7]Department of Field Crops, Faculty of Agriculture, Akdeniz University, Antalya, Türkiye; mehmettekin@akdeniz.edu.tr

*Correspondence: mehmettekin@akdeniz.edu.tr

Supplementary Materials

Table S1. A set of 70 bread wheat varieties, released from 1968 to 2022 in Türkiye, and their information

No	Variety	Release year	Registration holder*	No	Variety	Release year	Registration holder*
1	Bezostaja-1	1968	MAEM	36	Altındane	2012	KTAEM
2	Kıraç 66	1970	GKTAEM	37	Rumeli	2012	Trakya Tarım
3	Cumhuriyet 75	1976	ETAEM	38	Quality	2012	Ata Tohumculuk
4	Gerek 79	1979	GKTAEM	39	Vittorio	2012	Progen A.Ş.
5	Atay-85	1985	GKTAEM	40	Adelaide	2013	Maro Tarım A.Ş.
6	Kate A-1	1988	TTAEM	41	Avorio	2013	Maro Tarım A.Ş.
7	Dağdaş 94	1994	BDUTAEM	42	Dinç	2013	GAPUTAEM
8	Sultan 95	1995	GKTAEM	43	Gökkan	2013	DAKTAEM
9	Kaşifbey 95	1995	ETAEM	44	Segor	2013	Agro Teknik A.Ş.
10	İkizce 96	1996	TARM	45	Bora	2014	Tasaco Tarım A.Ş.
11	Pamukova 97	1997	MAEM	46	Genesi	2014	Tasaco Tarım A.Ş.
12	Pehlivan	1998	TTAEM	47	Glosa	2014	Tareks A.Ş.
13	Ziyabey 98	1998	ETAEM	48	Masaccio	2014	Progen A.Ş.
14	Gönen 98	1998	ETAEM	49	Nevzatbey	2014	KTAEM
15	Karacadağ 98	1998	GAPUTAEM	50	Tekin	2014	GAPUTAEM
16	Ceyhan 99	1999	DAKTAEM	51	Yakamoz	2014	DAKTAEM
17	Flamura 85	1999	Tareks A.Ş.	52	Efe	2015	ETAEM
18	Karahan-99	1999	BDUTAEM	53	Kale	2015	GAPUTAEM
19	Bayraktar 2000	2000	TARM	54	Leuta	2016	Tareks A.Ş.
20	Demir 2000	2000	TARM	55	Yüksel	2016	TTAEM
21	Sönmez 2001	2001	GKTAEM	56	Duru 17	2017	Tareks A.Ş.
22	Alparslan	2001	DATAEM	57	Hüseyinbey	2017	13 Yıldız Grup Tarım ve Tohum A.Ş.
23	Pandas	2001	DAKTAEM	58	Albachiara	2018	Tarım Kredi Tohumculuk A.Ş.
24	Sagittario	2001	TASACO Tarım A.Ş.	59	Damla	2018	TTAEM
25	Canik 2003	2003	KTAEM	60	Koç 2015	2018	BATEM
26	Tosunbey	2004	TARM	61	Anafarta	2019	TTAEM
27	Ahmetağa	2004	BDUTAEM	62	Abide	2019	TTAEM

28	Krasunia Odes'ka	2008	Marmara Tohum	63	Izvor	2019	Tarım Kredi Tohumculuk A.Ş.
29	Aldane	2009	TTAEM	64	Eylül	2020	TTAEM
30	Kenanbey	2009	TARM	65	Albaşak	2021	TTAEM
31	Selimiye	2009	TTAEM	66	Beyaz 1	2021	13 Yıldız Grup Tarım ve Tohum A.Ş.
32	ES 26	2010	GKTAEM	67	Boldane	2021	Tasaco Tarım A.Ş.
33	Esperia	2011	TASACO Tarım A.Ş.	68	Alba	2022	Tasaco Tarım A.Ş.
34	Cömert	2011	Avesa Tohumculuk	69	Shiro	2022	Tasaco Tarım A.Ş.
35	Aglika	2012	Tarar Un San.	70	Destra	2022 (production permitted)	Tasaco Tarım A.Ş.

*BATEM: Batı Akdeniz Agricultural Research Institute; BDUTAEM: Bahri Dağdaş International Agricultural Research Institute; DATAEM: East Anatolian Agricultural Research Institute; DAKTAEM: Eastern Mediterranean Agricultural Research Institute; ETAEM: Aegean Agricultural Research Institute; GAPUTAEM: GAP International Agricultural Research and Training Center; GKTAEM: Transitional Zone Agricultural Research Institute; KTAEM: Black Sea Agricultural Research Institute; MAEM: Maize Research Institute; TARM: Field Crops Central Research Institute; TTAEM: Thrace Agricultural Research Institute.

Table S2. Basic statistics, such as mean, minimum, maximum, coefficient of variation (CV), standard deviation (SD), skewness, and kurtosis, of reactions of bread wheat varieties to the *Pst* races

Race	Mean	Min	Max	CV (%)	SD	Skewness	Kurtosis
PSTr-27	7.67	4.00	9.00	14.67	1.13	-1.07^{**}	1.34^{*}
PSTr-28	6.61	4.00	8.00	16.54	1.09	-0.61^{*}	-0.27^{ns}
PSTr-29	6.63	3.00	8.00	15.66	1.04	-0.95^{**}	1.59^{*}
PSTr-30	6.61	3.00	8.00	15.51	1.03	-0.82^{**}	1.29^{ns}
PSTr-31	7.27	4.00	8.00	11.92	0.87	-1.80^{**}	4.57^{**}

$^{*}p<0.05$, $^{**}p<0.01$. ns: non-significant

Table S3. Molecular screening results obtained using markers linked to the *Yr5, Yr10, Yr15, Yr17, Yr18, Yr26, Yr36, Yr44,* and *YrSP* resistance genes*

Variety	*Yr5*	*Yr10*	*Yr15*	*Yr17*	*Yr18*	*Yr26*	*Yr36*	*Yr44*	*YrSP*
Bezostaja-1	-	-	-	-	+	-	+	-	-
Kıraç 66	-	+	-	-	+	-	-	-	-
Cumhuriyet 75	-	-	-	-	-	-	+	-	-
Gerek 79	-	-	-	-	-	-	-	-	-
Atay-85	-	-	-	-	-	-	-	-	-
Kate A-1	-	-	-	-	+	-	+	-	-
Dağdaş 94	-	-	-	-	+	-	+	-	-
Sultan 95	-	-	-	-	+	-	+	-	-

Kaşif Bey 95	-	-	-	-	+	-	+	-	-
İkizce 96	-	-	-	-	-	-	-	-	-
Pamukova 97	-	-	-	-	-	-	-	-	-
Pehlivan	-	-	-	-	+	-	+	-	-
Karacadağ 98	-	-	-	-	-	-	-	-	-
Gönen 98	-	-	-	-	+	-	+	-	-
Ziyabey 98	-	-	-	-	-	-	-	-	-
Karahan-99	-	-	-	-	+	-	-	-	-
Ceyhan-99	-	-	-	-	+	-	+	+	-
Flamura 85	-	-	-	-	+	-	+	-	-
Bayraktar 2000	-	-	-	-	-	-	-	-	-
Demir 2000	-	-	-	-	-	-	-	-	-
Sönmez 2001	-	-	-	-	+	-	+	-	-
Alparslan	-	-	-	-	-	-	+	-	-
Pandas (Panda)	-	-	-	-	-	-	+	-	-
Sagittario	-	-	-	-	-	-	+	+	-
Canik 2003	-	-	-	-	+	-	-	-	-
Tosunbey	-	-	-	-	-	-	+	-	-
Ahmetağa	-	-	-	-	-	-	+	-	-
Krasunia odes'ka	-	-	-	-	-	-	-	-	-
Kenanbey	-	-	-	-	+	-	+	-	-
Aldane	-	-	-	-	-	-	-	+	-
Selimiye	-	-	-	-	+	-	+	-	-
ES 26	-	-	-	-	-	-	+	-	-
Esperia	-	-	-	-	-	-	+	+	-
Cömert	-	-	-	-	+	-	+	-	-
Altındane	-	-	-	-	-	-	-	-	-
Vittorio	-	-	-	-	+	-	+	+	-
Quality	-	-	-	-	+	-	-	+	-
Rumeli	-	-	-	-	+	-	+	+	-
Aglika	-	-	-	-	+	-	+	+	-
Dinç	-	-	-	-	-	-	-	-	-
Gökkan	-	-	-	-	+	-	+	+	-
Segor	-	-	-	-	+	-	-	-	-
Adelaide	-	-	-	-	-	-	+	-	+
Avorio	-	-	-	-	+	-	+	+	-
Tekin	-	-	-	-	+	-	-	+	-
Nevzatbey	-	-	-	-	-	-	-	-	-
Yakamoz	-	-	-	-	-	-	+	-	-
Bora	-	-	-	-	+	-	+	+	-
Genesi	-	-	-	-	+	-	+	+	-
Glosa	-	-	-	-	-	-	-	-	-
Masaccio	-	-	-	-	-	-	-	-	-
Efe	-	-	-	-	-	-	-	-	-
Kale	-	-	-	-	-	-	-	-	-
Yüksel	-	-	-	-	-	-	-	-	-
Lenta	-	-	-	-	-	-	-	-	-
Duru	-	-	-	-	-	-	-	-	-

Hüseyinbey	-	-	-	+	-	-	-	-	-
Albachiara	-	-	-	-	-	-	-	-	-
Damla	-	-	-	-	-	-	-	-	-
Koç	-	-	-	-	-	-	-	-	-
İzvor	-	-	-	-	-	-	-	-	-
Anaforta	-	-	-	-	-	-	-	-	-
Abide	-	-	-	-	-	-	-	-	-
Eylül	-	-	-	-	-	-	-	-	-
Albaşak	-	-	-	-	-	-	-	-	-
Boldane	-	-	-	+	-	-	-	-	-
Beyaz-I	-	-	-	-	-	-	-	+	-
Shiro	-	-	-	+	-	-	-	-	-
Destra	-	-	-	-	-	-	-	-	-
Alba	-	-	-	-	-	-	-	-	-

*Presence (+) or absence (−) of the resistance genes based on the obtained molecular data

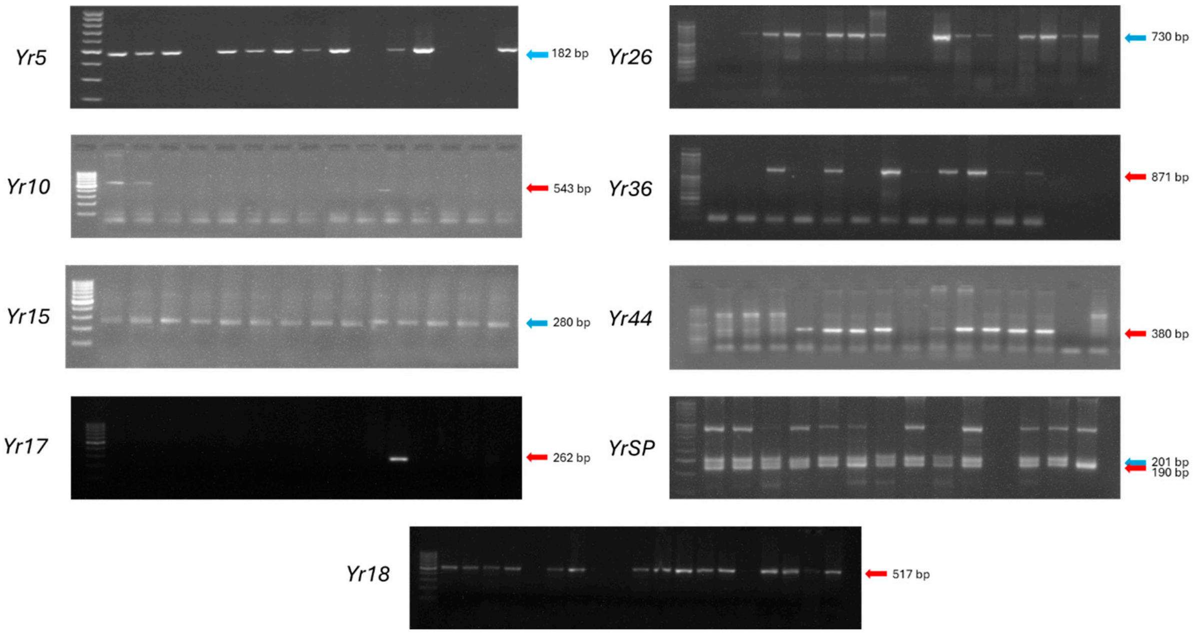

Figure S1. Agarose gel images of the molecular markers analyzed. Red arrows indicate bands containing resistance genes, while blue arrows indicate bands lacking resistance genes
